# The role of NLRP3 inflammasome in inflammation-related skeletal muscle atrophy

**DOI:** 10.3389/fimmu.2022.1035709

**Published:** 2022-11-03

**Authors:** Yukun Liu, Dongfang Wang, Tianyu Li, Fan Yang, Zhanfei Li, Xiangjun Bai, Yuchang Wang

**Affiliations:** ^1^ Department of Plastic and Cosmetic Surgery, Tongji Hospital, Tongji Medical College, Huazhong University of Science and Technology, Wuhan, China; ^2^ Trauma Center/Department of Emergency and Traumatic Surgery, Tongji Hospital of Tongji Medical College, Huazhong University of Science and Technology, Wuhan, China

**Keywords:** NLRP3, skeletal muscle, inflammasome, pyroptosis, metabolic syndrome, sepsis, ICUAW

## Abstract

Skeletal muscle atrophy is a common complication in survivors of sepsis, which affects the respiratory and motor functions of patients, thus severely impacting their quality of life and long-term survival. Although several advances have been made in investigations on the pathogenetic mechanism of sepsis-induced skeletal muscle atrophy, the underlying mechanisms remain unclear. Findings from recent studies suggest that the nucleotide-binding and oligomerisation domain (NOD)-like receptor family pyrin domain containing 3 (NLRP3) inflammasome, a regulator of inflammation, may be crucial in the development of skeletal muscle atrophy. NLRP3 inhibitors contribute to the inhibition of catabolic processes, skeletal muscle atrophy and cachexia-induced inflammation. Here, we review the mechanisms by which NLRP3 mediates these responses and analyse how NLRP3 affects muscle wasting during inflammation.

## Introduction

Skeletal muscle is a plastic organ and the most abundant tissue in vertebrates. It plays a key role in movement, respiration and metabolism. In the skeletal muscle of healthy individuals, there is a balance between protein synthesis and degradation. Critically ill patients in the ICU frequently experience substantial loss of muscle strength and mass, commonly known as intensive care unit-acquired weakness (ICUAW), which is associated with increased morbidity and mortality rates in these patients (1). Sepsis and systemic inflammation are the major risk factors for ICUAW (2, 3). It not only prolongs the ICU treatment time but also worsens the long-term prognosis of patients (1, 3, 4). However, the pathogenesis of inflammation-associated muscle atrophy remains unclear, hindering its diagnosis and treatment.

The nucleotide-binding and oligomerisation domain (NOD)-like receptor family pyrin domain containing 3 (NLRP3) inflammasome can detect various harmful stimuli, including pathogens such as bacteria and viruses and signals of tissue damage (5, 6). In the classical activation pathway, the NLRP3 inflammasome assembles and subsequently activates caspase-1 to induce pyroptosis, while proinflammatory cytokines such as interleukin (IL)-1β and IL-18 mature and are released, thus causing an inflammatory response (5, 6). Moderate activation of NLRP3 inflammasome can help the host effectively eliminate the microbial infection. However, excessive activation of the NLRP3 inflammasome causes excessive inflammation and cell damage (7–9). In recent years, NLRP3 has been reported to be widely involved in sepsis-related immune cell death and dysfunction of multiple organs (5, 7–10). Additionally, it is reportedly an important regulator of skeletal muscle metabolism (11–13). In recent times, an increasing number of studies have demonstrated that the NLRP3 inflammasome is involved in the pathogenesis and development of inflammation-related skeletal muscle wasting (14, 15). Here, we provide a comprehensive review of the current literature on the mechanisms and treatment of NLRP3 inflammasome in inflammation-related skeletal muscle depletion.

## Definition and composition of NLRP3 inflammasome

Tschopp, who first defined the inflammasome in 2002, revealed that inflammasomes play an important role in microbial infection, regulation of mucosal immune responses and metabolic processes (16). Inflammasome activation can also play an important role in pathogen defence by stimulating innate and adaptive immune responses (17). Inflammasome is a group of multiprotein complexes composed primarily of sensors, adapters and pro-caspase-1, which can recognise various stress, exogenous microorganisms and endogenous danger signals (18–21). NLRs include various isoforms, such as the NLRP1, NLRP3, NLRP6, NLRP7, NLRP12 and NLRC4 (22). NLRP3 inflammasome consists of NLRP3, adaptor apoptosis-related speck-like protein containing caspase recruitment domain (CARD) (ASC) and procaspase-1, which has been studied extensively (5, 9). NLRP3, as the core protein of the NLRP3 inflammasome, contains a central NOD (NACHT) that possesses ATPase activity and a propensity for self-oligomerise. When the host cell is stimulated by infection or other factors, NLRP3 inflammasome interacts with ASC through the CARD/CARD and pyrin domain (PYD)/PYD to catalyse the pre-cleavage of caspase-1 into two subunits, P20 and P10. Active caspase-1 is composed of P20 and P10 tetramers, which cleaves gasdermin D (GSDMD) to form activated N-GSDMD, which can perforate the cell membrane and induce programmed cell death, known as pyroptosis. Simultaneously, caspase-1 cleaves pro-IL-1β and pro-IL-18 to form IL-1β and IL-18, respectively, which are released from pyroptotic cells and initiate a cascade of pathological reactions (5, 9). At the same time, caspase-1 cleaves pro-IL-1β and pro-IL-18 to form IL-18 and IL-1β, which are released from pyroptotic cells and play a series of pathological reactions (23, 24). Dysregulation of NLRP3 inflammasome has been implicated in many human diseases, such as gout, diabetes and sepsis-related organ dysfunction and metabolic disorders (5, 25–28). Therefore, numerous inflammation-related diseases can be treated by targeting the NLRP3 inflammasome.

## Activation and regulation of NLRP3 inflammasome

There are two stages involved in the activation of the NLRP3 inflammasome. The first stage involves priming signals, such as Toll-like receptors (TLRs) and NLRs, which recognise specific pathogen-associated molecular patterns (PAMPs) or danger-associated molecular patterns (DAMPs) and activate nuclear factor kappa-B (NF-κB)-mediated upregulation of NLRP3 protein, IL-1β, and IL-18 expression (29). The second signal is the assembly of inflammasomes in response to the activation of PAMPs and DAMPs. NLRP3 assembles *via* the NACHT domain and provides a scaffold for ASC oligomerisation through the CARD homology interaction between PYDs and caspase-1. Activation of the NLRP3 inflammasome leads to self-cleavage of pro-caspase-1 to generate active caspase-1, which in turn mediates the maturation and secretion of IL-1β and IL-18. Additionally, activated caspase-1 can induce GSDMD-mediated pore formation, osmotic swelling and plasma membrane rupture, leading to a cascade of inflammatory reactions (30–33). This canonical NLRP3 inflammasome activation has been observed to occur in a variety of myopathies (11, 12, 34), including skeletal muscle atrophy caused by sepsis (14).

NLRP3 inflammasome can be activated by a variety of pathogenic and aseptic inflammatory signals (33, 35). Examples include exogenous PAMPs from fungi, bacteria and viruses, as well as host-derived molecules such as reactive oxygen species (ROS) and extracellular ATP. In addition, some crystals and particles (uric acid crystals, silica, asbestos and alum) are activated (36, 37). Lysosomal instability, mitochondrial function and ion flux dysfunction (K+ efflux, Ca2+ signalling, Na+ influx and Cl- efflux) are additional conditions that can activate the NLRP3 inflammasome (35). Multiple sources of Ca2+ lead to an increase in intracellular Ca2+ during NLRP3 inflammasome activation. The calcium-sensitive receptor (CaSR) and GPRC6A are stimulated and then activate phospholipase C, which then hydrolyzes phosphatidylinositol 4, 5-diphosphate (PIP2) to form inositol 1,4, 5-triphosphate (IP3) (38). IP3 then induces Ca2+ efflux from the lumen of the endoplasmic reticulum (ER) to the cytoplasm through ligand-gated ion channels, which are termed IP3 receptors (IP3R) (38). In addition, lysosomes have also been suggested to be an important source of Ca2+ and may contribute to NLRP3 inflammasome activation (39). Regardless of the source, this stimulation-induced increase in cytosolic Ca2+ was shown to be essential for NLRP3 inflammasome activation; however, how this increase in cytosolic Ca2+ contributing to NLRP3 inflammasome activation remains unclear. Furthermore, K+ efflux inducible stimuli can trigger NLRP3 inflammasome activation in macrophages when cultured with Ca2+ -free media, suggesting that at least the extracellular Ca2+ pool is not required for NLRP3 inflammasome activation (40, 41).

**Figure 1:**
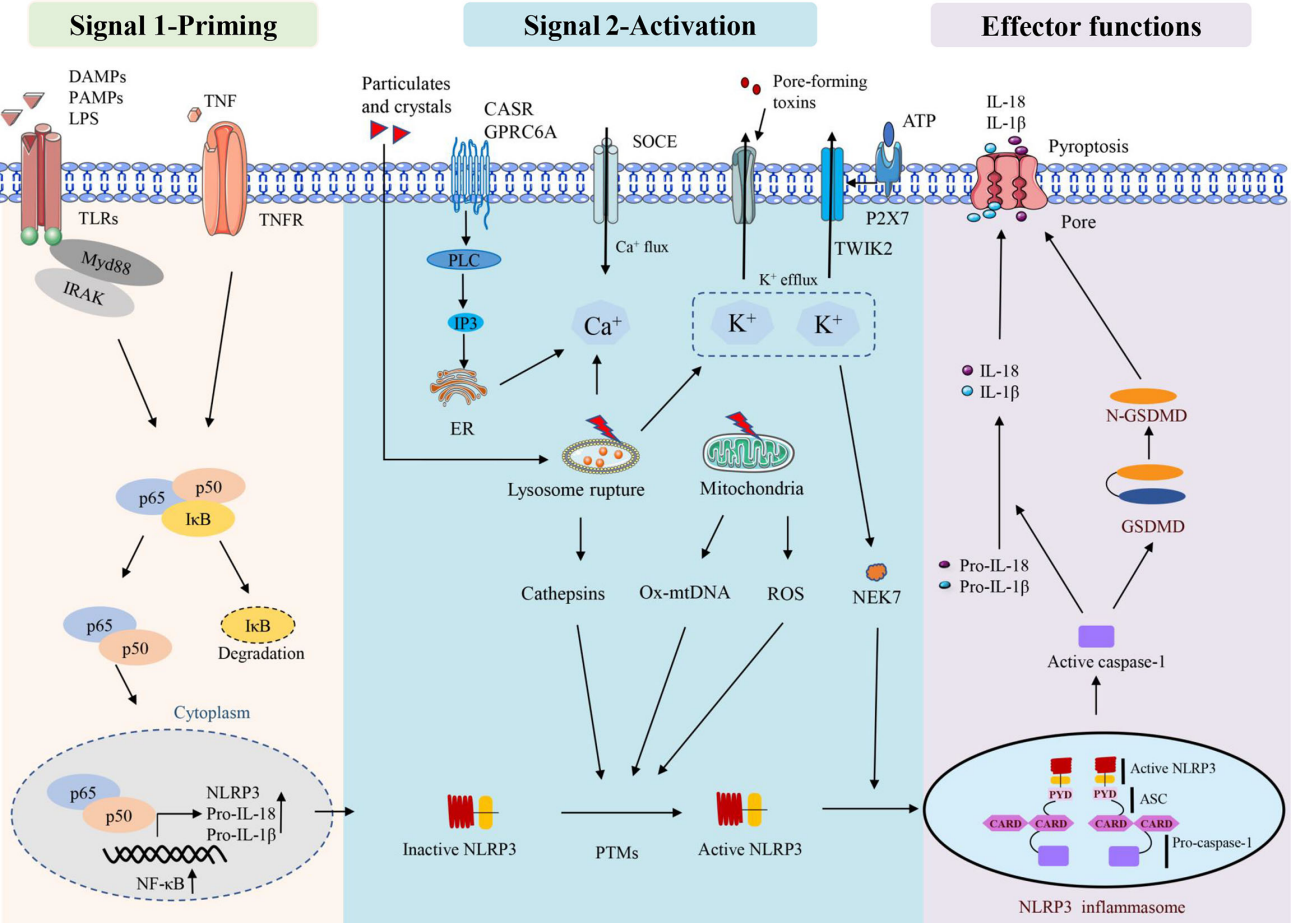
Activation and effector functions of the NLRP3 inflammasome.

ROS, especially mitochondrial ROS (mtROS), are important stimulators of NLRP3 activation (42–44). Mitochondrial dysfunction and ROS generation are important factors causing NLRP3 inflammasome activation, and ROS inhibitors or scavengers can limit inflammasome activation (45). In addition to mtROS, cytosolic mtDNA is a crucial factor mediating NLRP3 activation. Numerous NLRP3 activators can induce mtDNA release, and cytosolic oxidised mtDNA can trigger NLRP3 inflammasome assembly and activation (46). Oxidised mtDNA, a key component of the NLRP3 inflammasome, can directly interact with NLRP3 (47). NEK7, a member of the mammalian never in mitosis gene A (NIMA)-related kinase family (NEK protein), has been reported to bind to NLRP3, act downstream of potassium efflux and regulate NLRP3 oligomerisation and activation (48). NEK7 was observed to regulate gene transcription or protein expression in the NLRP3 inflammasome signalling pathway. These signalling pathways include ROS, potassium efflux, lysosomal destabilisation and NF-κB. In addition, NEK7 has been suggested as a potential therapeutic target for NLRP3-related diseases owing to its involvement in various NLRP3-related diseases in human or animal models (49). Mitochondria are thought to be the central organelle that regulates NLRP3 inflammasome activation. Mitochondrial destabilisation, NLRP3 deubiquitination, ASC linear ubiquitination and the externalisation or release of mitochondria-derived molecules such as cardiolipin and mtDNA. These molecules bind to mitochondrial translocated NLRP3 and activate NLRP3 inflammasomes (50) (**Figure 1**).

Some factors that disrupt lysosome function and homeostasis, including particle stimulation with alum, silicon and asbestos, cause lysosome rupture and release of particles into the cytoplasm to activate the NLRP3 inflammasome (51). Lysosomal content, such as cathepsin B, is thought to play a role in the initial assembly and activation of the inflammasome, which is also an important mechanism for particle activation of NLRP3 (52). CA-074-ME, a chemical inhibitor of cathepsin B, inhibits NLRP3 inflammasome activation through particulate matter (51, 53). It has been observed that lysosomal release of cathepsin B is required for the release of IL-1β, but not for pro-IL-1β production, which also suggests that cathepsin B is involved in NLRP3 inflammasome activation (39) (**Figure 1**).

NLRP3 can be regulated by various post-translational modifications, such as ubiquitination, phosphorylation and S-nitrosation (35). The activation of NLRP3 involves several regulators. For example, thioredoxin-interacting protein deficiency can affect the activation of the NLRP3 inflammasome, the secretion of IL-1β and improve glucose tolerance and insulin sensitivity (54). Guanylate binding protein 5 stimulates inflammasome assembly, promotes the selective response of NLRP3 inflammasome to pathogenic bacteria and soluble inflammasome primers and is considered a unique rheostat for NLRP3 inflammasome activation (55). In periodontal disease, double-stranded RNA (dsRNA)-dependent protein kinase R (PKR) regulates inflammation by regulating NLRP3 inflammasome surface through the NF-κB pathway (56) and migration inhibitor (57), microtubule affinity-regulating kinase 4 (58) and heat shock protein 90 (59, 60). Inhibition of NLRP3 involves multiple regulators, and the PYD-only protein 1 (POP1) inhibits ASC-dependent inflammasome assembly by preventing inflammasome nucleation, thereby interfering with caspase-1 activation and IL-1β and IL-18 release (61). The POP2 inhibits inflammasome assembly by binding to ASC and interfering with ASC recruitment to upstream sensors, thereby preventing caspase-1 activation and cytokine release (62). It can be concluded that many stimulators have involved in the activation and regulation of NLRP3, especially in the inflammatory microenvironment, while the underlying regulatory mechanisms still need to be further explored.

## NLRP3 and sepsis-induced muscle atrophy

Sepsis is an overreaction of the body to infection, leading to tissue and organ damage and muscle atrophy (63), which is a poor prognostic factor in sepsis (1). Different cell death types, including autophagy and necroptosis etc., are involved in skeletal muscle degradation or wasting (64, 65). Currently, excessive activation of NLRP3 inflammasome is found to be a significant factor in septic tissue inflammation and muscle atrophy (14, 66). *In vivo* and *in vitro* studies have confirmed that NLRP3-KO can reduce skeletal muscle atrophy caused by inflammation by reducing the expression of IL-1β (14). In the CLP mouse model, inhibition of the NLRP3/IL-1β pathway can alleviate sepsis-induced myocardial atrophy and cardiomyopathy and has a certain effect on the prevention of sepsis-induced cardiomyopathy (67). Furthermore, NLRP3/IL-1β, MuRF1 and MAFbx expression were significantly increased in mice with lipopolysaccharide (LPS)-induced sepsis. However, a dsRNA-dependent PKR inhibitor, a negative regulator of NLRP3, could inhibit the expression of these signals and significantly improve muscle atrophy and mass loss (68). Similarly, triptolide, a plant derivative that inhibits NLRP3 (69), attenuates LPS-induced myotube atrophy in C2C12 cells *in vitro*. It has a protective effect on the loss of skeletal muscle weight, strength and exercise ability and muscle atrophy induced by LPS in mice (70). In addition, dapansutrile (OLT1177) can inhibit NLRP3-ASC and the interaction of NLRP3-caspase-1, thereby inhibiting the oligomerisation of NLRP3 inflammasomes. It has also been demonstrated to reduce the IL-1β and oxidative stress induced by LPS in muscle and reverse metabolic consumption (71). Ketone body β-hydroxybutyrate (β-OHB) inhibits NLRP3 inflammasome by preventing K+ effusion and reducing ASC oligomerisation and spot formation (72). In humans, administration of the ketone body β-OHB reduces muscle protein breakdown following LPS injection. This indicates that β-OHB may have a protective effect against inflammation-induced muscle wasting (73). It can be concluded that NLRP3 is involved in inflammation-induced skeletal muscle atrophy and plays a central role. However, it should be emphasized that, to the best of our knowledge, no studies exploring the role of NLRP3-targeted drugs in septic myopathy exist. Therefore, this is an area of research that deserves further study.

## NLRP3 direct inhibitors

NLRP3 inflammasome is a potential therapeutic target for a variety of inflammatory diseases. Based on different mechanisms of action, we reviewed the NLRP3 inflammasome direct inhibitors that have been instigated in inflammatory diseases.

### Inflammasome assembly

The ketone body β-OHB inhibits the NLRP3 inflammasome by preventing K(+) efflux, reducing ASC oligomerisation and speck formation and protecting against muscle protein catabolism in volunteers with LPS-stimulated inflammation (72, 73). Exogenous hydrogen sulphide can reduce hyperglycaemia-induced fibrosis of diabetic diaphragm and enhance its biomechanical properties, possibly by inhibiting the inflammatory response mediated by nucleotide binding NLRP3 inflammasome (74, 75). RRx-001, which is currently considered to be a highly selective NLRP3 inhibitor, binds covalently to cysteine 409 of NLRP3 and blocks NLRP3-NEK7 interaction, thereby preventing the assembly of inflammasome (76). Fluoxetine, an FDA-approved drug for clinical depression, prevents NLRP3-ASC activation (77). INF39 is a non-toxic and irreversible acrylate NLRP3 inhibitor that inhibits NEK7-NLRP3 interaction, and subsequently inhibits NLRP3-NLRP3, NLRP3-ASC, ASC oligomerisation and speck formation interaction (78). Oridonin (Ori), a bioactive ent-kaurane diterpenoid, forms a covalent bond with the cysteine 279 of NLRP3 in NACHT domain to block the interaction between NLRP3 and NEK7, thereby inhibiting NLRP3 inflammasome assembly and activation (79). It can be concluded that NLRP3 is involved in inflammation-induced skeletal muscle atrophy and plays a central role. However, it should be emphasized that, to the best of our knowledge, no studies have explored the role of NLRP3-targeted drugs in septic myopathy yet. Therefore, research regarding to this area deserves to be further studied.

### Targeting the ATPase activity of NLRP3

To inhibit the ATPase activity of the NLRP3 inflammasome, several inhibitors have been developed. These include gleazone (CY-09), 3, 4-methylenedioxy-β-nitrostyrene (MNS), MCC950 and OLT1177 (80–84). The diarylsulfonylurea-containing compound MCC950 (also known as CP-456773), which directly targets the NACHT domain of NLRP3 and maintains NLRP3 in an inactive state, is one of the most extensively researched compounds (85–87). In phase II clinical trials for rheumatoid arthritis, MCC950 was observed to cause liver injury by increasing serum liver enzyme levels (88). MCC950 promotes glucose transporter type 4 translocation in skeletal muscle, reduces NLRP3 inflammasome activation in skeletal muscle and improves insulin resistance in obesity (89). Additionally, in the mouse model of valosin-containing protein (VCP) myopathy, MCC950 improved the physical performance of mice by inhibiting the activation of the NLRP3 inflammasome, which has an effective therapeutic potential in the treatment of VCP-related myopathy (90). Preliminary test results of MCC950 for Duchenne muscular dystrophy (DMD) pathogenesis were promising and also exhibited improved muscle performance and protection against muscle inflammation (91). Thus, MCC950 can be a promising treatment option for a variety of myopathies.

OLT1177 is believed to covalently modify the NACHT domain to block its ATPase activity, ameliorate systemic and muscle inflammation and reduce muscle wasting in LPS-stimulated mice (71). Compound 6, a tetrahydroquinoline inhibitor of the NLRP3 inflammasome, was recently discovered and synthesised. It inhibits NLRP3 inflammasome assembly and activation by directly binding to the NACHT domain, inhibiting its ATPase activity and preventing ASC oligomerisation (92). CY-09 directly binds to the ATP-binding motif of the NLRP3 NACHT domain to inhibit its activity (93). NLRP3 ATPase activity is also disrupted by direct binding to MNS (94) and several other compounds, including BOT-4-one (95) and INF39 (78) (**Table 1**). Current available data suggest that several reagents targeting NLRP3 ATPase activity have protective effects against skeletal muscle inflammation and failure. However, its protective effect on sepsis-induced skeletal muscle wasting, especially in clinical trials, needs to be further verified.

**Table 1:** Direct NLRP3 inhibitors and their mechanisms.

Agent	Mechanism	Cell or animal model	Ref.
MNS	Inhibitory of NLRP3 ATPase activity	Bone-marrow derived macrophages	(94)
CY-09		Monocytes; mouse models of cryopyrin-associated autoinflammatory syndrome (CAPS) and type 2 diabetes	(93)
MCC950		iPSC-Derived VCP Patient Myoblasts; VCPR155H/+ Mice;mouse model of Duchenne muscular dystrophy	(12, 86, 90, 91)
OLT1177		LPS-stimulated human blood-derived macrophages; mouse model of LPS-induced systemic inflammation	(71)
INF39		Macrophages	(78)
Compound 6		Dextran sulfate sodium (DSS)-induced colitis mouse model	(92)
BOT-4-one		Bone-marrow derived macrophages primed with LPS	(95)
Fluoxetine		Alu RNA-induced RPE degeneration in mice	(77)
β-OHB	Inhibitory of NLRP3 oligomerization	Human monocytes were stimulated with LPS; Mouses were primed with LPS	(72)
RRx-001		RRx-001 ameliorates inflammatory diseases by acting as a potent covalent NLRP3 inhibitor	(76)
Tranilast		BMDMs from C57BL/6 mice; mouse models of gouty arthritis, cryopyrin-associated autoinflammatory syndromes, and type 2 diabetes	(96)
Oridonin		BMDMs treated with 50 ng/ml LPS	(79)

## NLRP3 indirect inhibitors

### Target upstream signals

Blocking the ATP receptor P2X7 is one potential method that researchers have tried to inhibit the NLRP3 inflammasome. Avastin is a P2X7 receptor (P2X7R) selective inhibitor that can prevent ATP-induced NLRP3 inflammasome activation (97). However, studies have reported that P2X7 stimulation can improve the innervation and metabolism of muscle fibres in amyotrophic lateral mice models and induce the proliferation/differentiation of satellite cells. Therefore, skeletal muscle denervation is prevented (98). Additionally, by blocking P2X7/K+ channels, both bright blue G (99–101) and Glyburide (101) demonstrated a recovery of muscle strength in IIM mouse models. MM01 interferes with ASC particle formation and oligomerisation, which prevents procaspase-1 activation *in vitro* and inhibits ASC-dependent inflammasome activation in cell lines (77, 102). IC100, a novel humanised antibody targeting ASC, has been demonstrated to be effective in preventing and/or suppressing the disease in an experimental autoimmune encephalomyelitis model (103).

Several natural extracts have exhibited remarkable potential in the treatment of inflammatory diseases. Triptolide inhibits NF-κB/TNF-α and regulates protein synthesis/degradation pathways to prevent LPS-induced skeletal muscle atrophy (70). Carbenoxolone improves insulin sensitivity in high-fat diet-induced obese mice by regulating the NLRP3 inflammasome (104). Melatonin has been demonstrated to improve muscle structure and activity in sarcopenic mice (11, 105). Curcumin was demonstrated to reduce ROS levels and proinflammatory cytokines in C2C12 muscle cells with palmitate-induced inflammation. It was also reported to improve the dystrophic phenotype in muscular dystrophy X-linked (MDX) mice (106, 107). In cell cultures and animal models, molecules such as adiponectin, metformin and resveratrol have also been observed to attenuate DMD, primarily through activation of AMP-activated protein kinase signalling and limiting inflammasome activation (108–110). Inflammasome NLRP3 expression is upregulated in DMD skeletal muscle fibers, where it is downregulated by ApN and its anti-inflammatory mediator Mir-711 and attenuates the dystrophic phenotype, suggesting that NLRP3 inhibitors may have therapeutic potential for muscle inflammation and myopathy (111). In addition, ghrelin was reported to improve motor function, attenuate muscle damage and reduce inflammatory cell infiltration in MDX mice through NLRP3 inflammasome activation (112). Shikenin, a pyruvate kinase M2 inhibitor used in Chinese medicine, inhibits NLRP3 activation and protects muscle cells (113, 114). Human volunteers on a high-palmitate diet (saturated fatty acids) had high levels of NLRP3 mRNA in skeletal muscle biopsies, whereas switching to a high-oleate diet (monounsaturated fatty acids) reduced NLRP3 priming and activation (115). Trimetazidine attenuates dexamethasone-induced muscle atrophy by inhibiting NLRP3/GSDMD pathway-mediated pyrosis (116). Although indirect inhibitors can prevent NLRP3 inflammasome activation, some of these molecules may have a tissue-specific mechanism of action. Additionally, the alkaloid piperlongumine (PL) from *Piper Longum* L. can prevent NLRP3 activity by interfering with the assembly of NLRP3 and NEK7 and NLRP3 oligomerisation (117). Licochalcone B, a major component of liquorice, directly binds to NEK7 and inhibits the interaction between NLRP3 and NEK7, thereby inhibiting the activation of NLRP3 inflammasome (118). Andrographolide, a bioactive chemical in andrographolide, inhibits NLRP3 activation by promoting mitophagy (119, 120). Other recently identified herbaceous agents include brevilin A (121), pristimerin (Pri) (122), pterostilbene derivatives (123) and berberine (124), all of which exhibit limiting effects on NLRP3 inflammasome activation. Some drugs have certain protective effects on skeletal muscle atrophy under certain conditions, but whether they play a protective effect on sepsis-induced skeletal muscle depletion remains to be discussed. There is no doubt that these drugs, especially natural extracts, offer broader ideas for the treatment of skeletal muscular atrophy because of their regulatory effects on NLRP3.

### Targeting downstream signals

Several drugs and molecules may act downstream of the NLRP3 inflammasome to inhibit pyroptosis and/or inflammation. The downstream signals of NLRP3 include caspase-1, IL-1β/IL-1R and IL-18. Caspase-1 inhibitors include ritonavir, disulfiram and VX-765 (125–127). As previously described, disulfiram and VX-765 act to improve the prognosis of sepsis by blocking the formation of GSDMD pores (128); however, whether it improves skeletal muscle metabolism remains unclear. Anti-IL-1β therapies were first tested in humans and showed efficacy in several inflammatory diseases albeit their effects on metabolic disorders are less significant (129, 130). Anti-IL-18 therapies are currently being developed for different inflammatory diseases. For example, a humanised antibody to IL-18, GSK1070806, is currently being assessed in phase I trials in atopic dermatitis (ClinicalTrials.gov Identifier: NCT04975438). Recent studies have demonstrated that dimethyl fumarate can react with key cysteine residues of GSDMD to form S-(2-succinyl)-cysteine, thereby inhibiting GSDMD-induced cell death (131). Surprisingly, several drugs targeting the downstream of NLRP3 have conducted clinical trials for certain diseases, and to our knowledge, these drugs have not been investigated for the treatment of sepsis-related skeletal muscle atrophy yet. Therefore, it is an urgent demand of developing new therapies that directly targeting the NLRP3 inflammasome.

## Conclusion and future perspectives

It has been more than 20 years since NLRP3 inflammasome was first discovered. With overwhelming studies have been conducted in these years, we’ve gained comprehensive understanding of the structure, composition, regulation, and function of NLRP3. However, the precise molecular mechanism of NLRP3 regarding to diseases has not been fully elucidated. In recent years, the role of NLRP3 inflammasome in skeletal muscle wasting has drew growing attention. Increasing evidence has confirmed that NLRP3 inflammasome activation plays an important role in the pathogenesis and progression of inflammation-related skeletal muscle wasting. In both cellular and animal models, inhibition of NLRP3 body assembly or activation can alleviate skeletal muscle atrophy and thereby enhance muscle strength. Therefore, targeting the NLRP3 inflammasome may represent a new trend in inflammation-related skeletal muscle wasting. The activation and regulation of NLRP3 inflammasome involves upstream signal-related initiation signals, activation signals, regulatory factors, and downstream caspase-1, IL-1β and IL-18. Currently, strategies to block downstream inflammatory cytokines, such as inhibitors targeting IL-18, have been used in clinical trials, but the results remain unknown. At present, attention has gradually turned to NLRP3 inflammasome and their constituent molecules, and many targeted drugs have been developed for the purpose of maximizing therapeutic specificity and reducing nonspecific effects. In addition, although upstream regulators of NLRP3 inflammasome are also considered as promising pharmacological targets, their interactions are not specific. To date, although many compounds have been found to exert regulatory effects on NLRP3 inflammasome *in vivo* or *in vitro*, their therapeutic efficacy and safety in patients with skeletal muscle wasting need to be further verified in clinical trials. In addition, it is surprising that more and more traditional Chinese herbal medicines and plant-derived compounds have been found to be effective and safety, and they are expected to provide new direction for the treatment of skeletal muscle wasting.

In conclusion, NLRP3 inflammasome overactivation plays a key pathological role in the development and progression of sepsis-induced skeletal muscle atrophy. As we continue to comprehend the physiological and pathological mechanisms involved and the development of new therapies targeting the NLRP3 inflammasome, promising outcomes have been demonstrated in animal studies. Several NLRP3 inhibitors have been approved for the use in human clinical trials, and it is believed that the treatment and the drug development targeting NLRP3 will provide new directions for the prevention and strategies of sepsis-induced muscle atrophy.

## Data availability statement

The raw data supporting the conclusions of this article will be made available by the authors, without undue reservation.

## Author contributions

All authors contributed to the article and approved the submitted version. YL, DW and TL undertook the research, YL and FY wrote the main manuscript text and prepared figures. ZL, XB and YW revised the article critically for important intellectual content and final approval of the version to be submitted.

## Funding

This study was supported by grants from the National Natural Science Foundation of China (Grant No. 82002101,82002096).

## Acknowledgments

We would like to thank the reviewers for their helpful comments on this article.

## Conflict of interest

The authors declare that the research was conducted in the absence of any commercial or financial relationships that could be construed as a potential conflict of interest.

## Publisher’s note

All claims expressed in this article are solely those of the authors and do not necessarily represent those of their affiliated organizations, or those of the publisher, the editors and the reviewers. Any product that may be evaluated in this article, or claim that may be made by its manufacturer, is not guaranteed or endorsed by the publisher.
